# The Preparation, Characterization and Formation Mechanism of a Calcium Phosphate Conversion Coating on Magnesium Alloy AZ91D

**DOI:** 10.3390/ma11060908

**Published:** 2018-05-28

**Authors:** Dong Liu, Yanyan Li, Yong Zhou, Yigang Ding

**Affiliations:** 1 School of Chemistry and Environmental Engineering, Wuhan Institute of Technology, Wuhan 430205, China; liudong@wit.edu.cn (D.L.); lyy920313@126.com (Y.L.); 2 Key Lab for Green Chemical Process of Ministry of Education, Wuhan Institute of Technology, Wuhan 430205, China; zhouyong@wit.edu.cn; 3 State Key Laboratory of Oil and Gas Reservoir Geology and Exploitation, Southwest Petroleum University, Chengdu 610500, China

**Keywords:** magnesium alloy, calcium phosphate coating, corrosion resistance, SEM, formation mechanism

## Abstract

The poor corrosion resistance of magnesium alloys is one of the main obstacles preventing their widespread usage. Due to the advantages of lower cost and simplicity in operation, chemical conversion coating has drawn considerable attention for its improvement of the corrosion resistance of magnesium alloys. In this study, a calcium phosphate coating was prepared on magnesium alloy AZ91D by chemical conversion. For the calcium phosphate coating, the effect of processing parameters on the microstructure and corrosion resistance was studied by scanning electron microscope (SEM) and electrochemical methods, and the coating composition was characterized by X-ray diffraction (XRD). The calcium phosphate coating was mainly composed of CaHPO_4_·2H_2_O (DCPD), with fewer cracks and pores. The coating with the leaf-like microstructure provided great corrosion resistance to the AZ91D substrate, and was obtained under the following conditions: 20 min, ambient temperature, and no stirring. At the same time, the role of NH_4_H_2_PO_4_ as the coating-forming agent and the acidifying agent in the conversion process was realized, and the formation mechanism of DCPD was discussed in detail in this work.

## 1. Introduction

Due to their low density, high strength/weight ratio, good machinability, and excellent recyclability, magnesium (Mg) and its alloys are becoming increasingly popular for use in various industries, including transportation, communication, and personal electronics [1,2,3,4]. Unfortunately, the poor corrosion and high chemical reactivity of Mg and its alloys, especially in chloride-containing environments, restrict their widespread usage [5,6,7,8]. Hence, it is critical to develop an effective protection technology in order to enhance the corrosion protection to magnesium and its alloys. At present, anodizing [9,10], micro-arc oxidation [11], chemical conversion coatings [12,13,14,15], and electroless plating [16] are the common surface treatments for improving the corrosion resistance of magnesium alloys, of which phosphate conversion coatings are regarded as one of the most effective and cheapest methods [17,18]. Up to now, a series of phosphate conversion coatings containing zinc-based [19], manganese-based [20], barium-based [21], and calcium-based phosphate conversion coatings [22,23] has been developed. Due to the intrinsic bioactivity and biocompatibility of calcium-based coating, and the environmentally friendly conversion technology leading to widespread application in the medical field [24,25], calcium-based phosphate conversion coating is becoming the hot topic in surface treatments of magnesium alloys. Kumta et al. [26] reported that a calcium phosphate coating was deposited on AZ31 magnesium alloy through acid etching, after which it was rinsed, polished, subjected to sonication and alkaline treatment, and finally immersed for 72 h at 37 °C. However, this pretreatment process and immersion time are too intricate and too time consuming to become popular. Su et al. [27,28] selected the ternary system containing Ca(NO_3_)_2_, CaO, and H_3_PO_4_ to deposit the CaHPO_4_·2H_2_O coating on AZ61 magnesium alloy at the necessary pH adjusted by NaOH solution. The problem is that the difficulty of preparing the calcium phosphate bath and the given temperature still hinders the industrialization of the process. Additionally, Song et al. [29,30] developed a simple calcium phosphate solution consisting of Ca(NO_3_)_2_ and NH_4_H_2_PO_4_. Although the phosphate conversion coating showed excellent corrosion resistance for Mg-8.8Li alloy, the operation conditions needed the specific temperature and pH, as well as complicated pretreatments including grinding, ultrasonic cleaning, polishing with 0.5 μm diamond paste, and etching. On this basis, Sun et al. [31] optimized this process and studied the effect of the addition of triethanolamine on the morphology and corrosion resistance of calcium phosphate coating. However, the pretreatment was still complex, and the calcium phosphate bath needed the HNO_3_/NaOH solution to adjust the appropriate pH value. Hence, it is urgent to develop a calcium phosphate solution with simple components that is convenient to prepare, and a short conversion coating process at room temperature.

In this work, the binary system including Ca(NO_3_)_2_·4H_2_O and NH_4_H_2_PO_4_ is selected as the coating-forming agent. Meanwhile, NH_4_H_2_PO_4_ acts as an acidifying agent for optimizing the composition ratio of the calcium phosphate solution. In addition, the effect of processing parameters, such as conversion time, temperature, pH, and stirring rate, on the morphology and corrosion resistance of conversion coating is investigated by means of SEM, potentiodynamic polarization curve, and electrochemical impedance spectroscopy (EIS). Finally, the formation mechanism is also proposed to reveal the changes of coating in the process of deposition.

## 2. Materials and Methods

The experimental material used for this investigation is die-casting AZ91D magnesium alloy (10 mm × 10 mm × 18 mm) with a nominal composition (wt %) of 9.1% Al, 0.9% Zn, 0.24% Mn, 0.031% Si, 0.0023% Fe, 0.015% Cu, 0.0005% Ni, and Mg balance. The specimens were connected to lead wires and embedded in epoxy resins with an exposed surface of 1 cm^2^. Before treatment, the sealed electrode was carefully polished with 2000-grit SiC papers, rinsed with water, and then dried in cold air.

Various calcium phosphate (Ca-P) solutions containing 25 g/L Ca(NO_3_)_2_·4H_2_O and different concentrations of NH_4_H_2_PO_4_, i.e., 5 g/L, 10 g/L, 15 g/L, 20 g/L and 25 g/L NH_4_H_2_PO_4_, were prepared for obtaining different Ca(NO_3_)_2_·4H_2_O:NH_4_H_2_PO_4_ concentration ratios of Ca-P solution. Hence, the corresponding concentration ratios of the Ca-P solution are 5:1, 5:2, 5:3, 5:4, and 5:5, respectively. Meanwhile, the corresponding pH values of the different concentration ratios of Ca-P solution are 3.5, 3.0, 2.8, 2.6, and 2.5 respectively. When the concentration ratio of Ca(NO_3_)_2_·4H_2_O:NH_4_H_2_PO_4_ is 5:1 and 5:2, the Ca-P solution presents clear and transparent. When it comes to the concentration ratio of 5:3, the status of the Ca-P solution presents as slightly turbid, indicating the maximum solubility of Ca(NO_3_)_2_·4H_2_O and NH_4_H_2_PO_4_ under such conditions. However, the further increase in concentration ratio (5:4) leads to the occurrence of precipitations in such Ca-P solution, and more precipitations appeared in the higher concentration ratio of Ca-P solution (5:5).

To obtain the optimum processing parameters, the pre-treated AZ91D magnesium samples were immersed in Ca-P baths with different Ca(NO_3_)_2_·4H_2_O:NH_4_H_2_PO_4_ concentration ratios, temperatures, immersion times, pH values, and stirring rates, and were then studied by electrochemical tests to evaluate the corrosion resistance of the coating. The corrosive medium was 3.5% (wt %) NaCl. Electrochemical tests were carried out using a classical three electrodes cell with platinum as the counter electrode, saturated calomel electrode (SCE) as the reference electrode, and the AZ91D specimen as the working electrode. The potentiodynamic polarization curves were obtained using an electrochemical analyzer (CS 310, Wuhan, China) at a constant voltage scan rate of 1 mV/s from −200 mV_OCP_ to an anodic current density of 10^−3^ A/cm^2^. Electrochemical impedance spectroscopy (EIS) measurements were conducted at open circuit potential (OCP) over a frequency range of 100 kHz to 10 mHz with a sinusoidal amplitude of 5 mV. Prior to each electrochemical test, a stabilization period of 1800 s was applied.

The treated surface was characterized respectively by X-ray diffraction (XRD, D8, Karlsruhe, Germany) using a Cu Kα radiation, and by field-emission scanning electron microscopy (SEM, JSM-5510LV, Kyoto, Japan) equipped with an energy dispersion X-ray spectrometry (EDS).

## 3. Results and Discussion

### 3.1. Ca-P Bath Component

**Figure 1:**
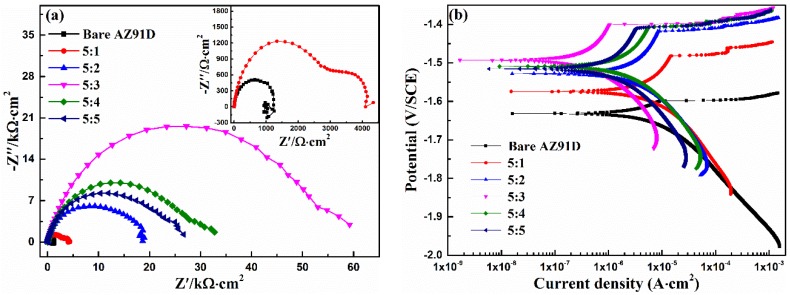
The electrochemical impedance spectroscopy (EIS) plots (**a**) and potentiodynamic polarization curves (**b**) of bare AZ91D alloy and the calcium phosphate coatings obtained from different concentration ratios of Ca-P solution in 3.5 wt % NaCl solution.

Generally, the calcium phosphate conversion takes place in acid solution, which needs HCl/NaOH solution to adjust the pH of the Ca-P solution. This process is complicated for the preparation of the Ca-P bath. In this study, Ca(NO_3_)_2_·4H_2_O (a nearly neutral substance) and NH_4_H_2_PO_4_ (an acidic substance) were selected as coating-forming agents. Meanwhile, NH_4_H_2_PO_4_ (as acidifying agent) with different masses was added into the 25 g/L Ca(NO_3_)_2_·4H_2_O solution, in order to reach the optimal pH value and optimal component ratio of the Ca-P bath to form high corrosion resistance of the calcium phosphate coating. The effect of Ca-P solution with different Ca(NO_3_)_2_·4H_2_O:NH_4_H_2_PO_4_ concentration ratios on the corrosion resistance of the coating was investigated by potentiodynamic polarization curve and EIS. Figure 1 shows the EIS plots and potentiodynamic polarization curves of bare AZ91D alloy and the calcium phosphate coatings obtained from different concentration ratios of Ca-P solution in 3.5% NaCl solution.

According to Figure 1a, the EIS plot of bare AZ91D shows one high frequency capacitive loop and one low frequency inductive loop accompanied with dispersive points, while there are two capacitive loops in the high and low frequencies existing in the EIS plot of concentration ratio 5:1. According to recent research, the appearance of low frequency inductive loops is mainly attributed to the fact that the oxidation of magnesium proceeds faster than hydrogen evolution at the corrosion front [32,33], and the accompanying dispersive points are related to the high activity of magnesium alloys [34]. The EIS plot indicates that the bare AZ91D alloy shows poor corrosion resistance, and the conversion coating formed in the concentration ratio 5:1 of Ca-P solution cannot provide enough protection effect. In addition, only one capacitive loop existed in the rest of the EIS plots, which indicates that the conversion coating is undamaged. Therefore, the conversion coating can remarkably improve the corrosion resistance of Mg substrate. In these EIS plots, the diameter of the capacitive loop is largest at the condition of concentration ratio 5:3, indicating that the conversion coating formed on AZ91D alloy at such a condition realizes its best corrosion protection performance.

**Figure 2:**
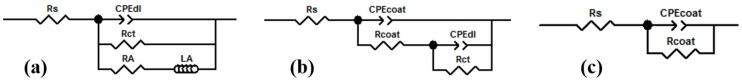
The equivalent electrical circuits used for the fitting of the EIS data: (**a**) magnesium substrate; (**b**) double capacitance loop and (**c**) single capacitance loop.

According to the corrosion process and referring to the literature [29,32,33,34,35,36], three equivalent circuits in Figure 2 are presented to fit the EIS results, where *R_S_* is the solution resistance, *R_ct_* is the charge-transfer resistance, *CPE_dl_* is the constant phase element of double layer at the metal/electrolyte, *R_coat_* is the conversion coating resistance, and *CPE_coat_* is the constant phase element of the coating, respectively. Finally, *L_A_* accounts for the variation of the extension of active anodic regions during the sinusoidal polarization, and *R_A_* represents the resistances associated with local environmental changes (precipitation of gels, presence of bubbles) in the vicinity of the anodic and cathodic regions [33]. The fitting parameters for Figure 1a are listed in Table 1, in which the higher *R_coat_* value represents better corrosion resistance [37,38,39]. In Table 1, it can be seen that the value of *R_coat_* representing the corrosion resistance of conversion coating increases with the increase of the concentration ratio. This is attributed to the fact that the increased content of NH_4_H_2_PO_4_ in the coating-forming agent ensures the occurrence of a coating-forming reaction by supporting enough coating-forming agent, and that the decreased pH value ensures the triggering of the corrosion reaction of the magnesium alloy to induce the alkaline environment near the surface of the magnesium alloy; this then accelerates the formation of the conversion coating, which also offers sufficient deposition sites, and thus enhances the adhesive force of the conversion coating [27,30,40]. The conversion coating provides the best protective performance for AZ91D alloy when the pH value and the concentration ratio of Ca-P solution equal 2.8 and 5:3, respectively. Meanwhile, in this case, there is no precipitation in the Ca-P solution, which realizes the optimum utilization of raw material. However, precipitation did appear in the Ca-P solution with a further increase of the concentration ratio, leading to the waste of raw material and the decrease in the values of pH and *R_coat_*. This is mainly attributed to the fact that the corrosion reaction of magnesium alloy surface induced by the decrease of pH becomes the control step suppressing the formation of conversion coating, and that the appearance of precipitation decreases the effective content of the coating-forming agent in the Ca-P solution. From the above, it can be seen that the AZ91D alloy immersed into the Ca-P solution with a concentration ratio of 5:3 and a pH of 2.8 obtains the best corrosion resistance, realizing the aims of being the lowest-cost option, and of having NH_4_H_2_PO_4_ act as the coating-forming agent and the acidifying agent.

Figure 1b shows the potentiodynamic polarization curves of the bare AZ91D alloy and the calcium phosphate coatings obtained from different concentration ratios of Ca-P solution in 3.5 wt % NaCl solution. It can be clearly seen that both the cathodic and anodic current density are depressed with the increase in concentration ratio, and the passivation behavior is observed at the anodic process of coated AZ91D alloy, suggesting that the conversion coating provides good protection for the magnesium substrate. Meanwhile, the corresponding electrochemical parameters, such as corrosion potential (*E_corr_*), corrosion current density (*i_corr_*), and cathodic Tafel slopes (*b_c_*) determined by the Tafel extrapolation method are listed in Table 2. According to Table 2, compared with the bare AZ91D alloy, the *E_corr_* of AZ91D alloy with the conversion coating presents the obvious positive shift, and the *E_corr_* at the concentration ratio of 5:3 is the most positive, implying a decrease in the tendency of corrosion initiation due to thermodynamics [35]. In addition, the corrosion current density of the conversion coating obtained from the concentration ratio of 5:3 is the lowest, and depressed two orders of magnitude compared with the corrosion current density of the bare AZ91D alloy, which is consistent with the results of EIS.

In short, when the ratio of Ca(NO_3_)_2_·4H_2_O to NH_4_H_2_PO_4_ is 5:3, the conversion coating realizes the best corrosion protection performance for the bare AZ91D alloy, where there is no precipitation in the Ca-P solution. Hence, it can ensure the minimum consumption of chemical reagent and the lowest cost, which is favorable for its extension to industrial production.

### 3.2. Conversion Time

**Figure 3:**
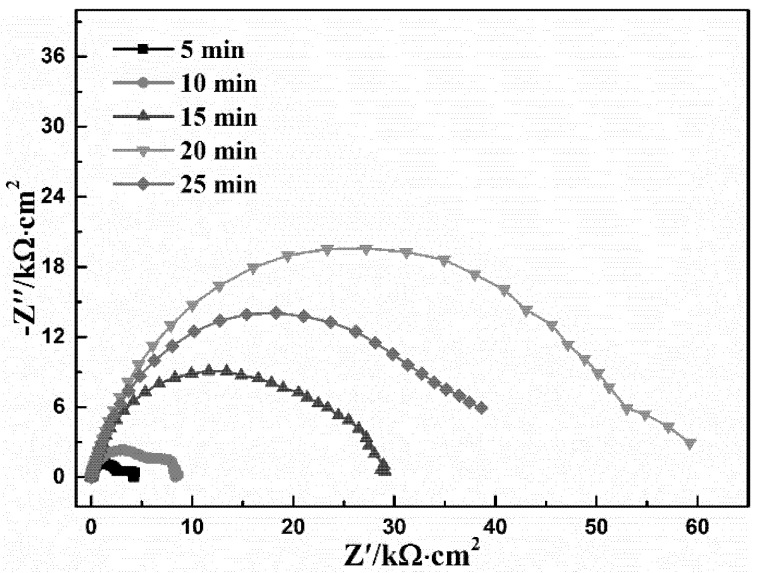
Experimental Nyquist plots of coated AZ91D samples obtained from different conversion times.

Too short a conversion time will result in the incomplete conversion coating [27]; however, too long a conversion time will again contribute to the dissolution of the conversion coating [41], which decreases its corrosion resistance. Hence, conversion time is an important factor influencing the corrosion resistance of the conversion coating. As shown in Figure 3, the diameter of the capacitive loop increases with the prolongation of time up to 20 min, however the diameter of the capacitive loop decreases after 20 min, which indicates that 20 min is the optimal conversion time. The *R_coat_* fitted by the equivalent electrical circuit in Figure 2 is listed in Table 3. From Table 3, it can be seen that the *R_coat_* at 20 min is the biggest, further indicating that 20 min is the optimal conversion time, obtaining the best protective performance. However, with further prolongation of the conversion time, the value of *R_coat_* decreases, suggesting that the compact conversion coating formed on the surface of the AZ91D alloy may partially dissolve.

**Figure 4:**
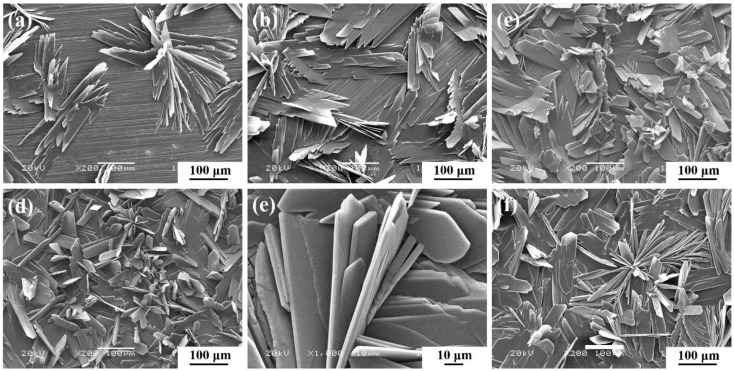
SEM micrographs of the calcium phosphate coatings under different conversion times: (**a**) 5 min; (**b**) 10 min; (**c**) 15 min; (**d**) 20 min; (**e**) magnification of 20 min and (**f**) 25 min.

In order to further understand the above results, the SEM of the conversion coating at different times was performed as shown in Figure 4. According to the results shown in Figure 4, it is obvious that the leaf-like conversion coating gradually covers the whole surface of AZ91D, and the scratches become increasingly indiscernible from 5 min to 20 min. However, when the immersion time increases to 25 min, as shown in Figure 4f, the coating formed at 25 min is sparser and less compact than the coating formed at 20 min. It is apparent that the coating formed at 20 min is the most compact, and thus presents the best protection for bare AZ91D, which is in accordance with the analysis of EIS.

### 3.3. Conversion Temperature

**Figure 5:**
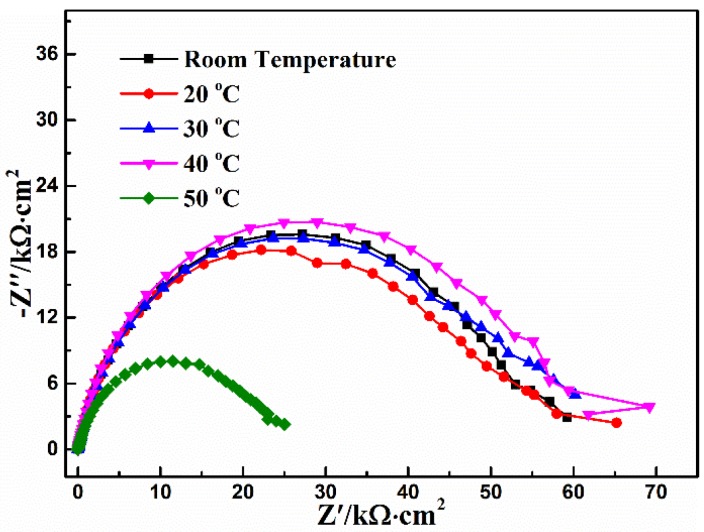
Experimental Nyquist plots of coated AZ91D samples obtained from different conversion temperatures.

The EIS of the conversion coating obtained from different temperatures of Ca-P solution is presented in Figure 5. The fitting results according to Figure 2 are listed in Table 4. The value of *R_coat_* basically keeps a similar level between 20 °C and 40 °C, while the *R_coat_* decreases drastically at 50 °C, indicating that high temperature decreases the corrosion resistance of the conversion coating. This phenomenon is mainly attributed to the fact that high temperature will generate a corrosion rate higher than the deposition rate of the conversion coating, and will also accelerate the mass diffusive rate of the species of deposition reaction, which leads to a decrease in probability of coating deposition. Meanwhile, the hydrogen evolution and bubble bursting occurred more easily at a high temperature, thus making the phosphate crystals difficult to nucleate [27] and decreasing the corrosion resistance of the coating.

### 3.4. pH Value

**Figure 6:**
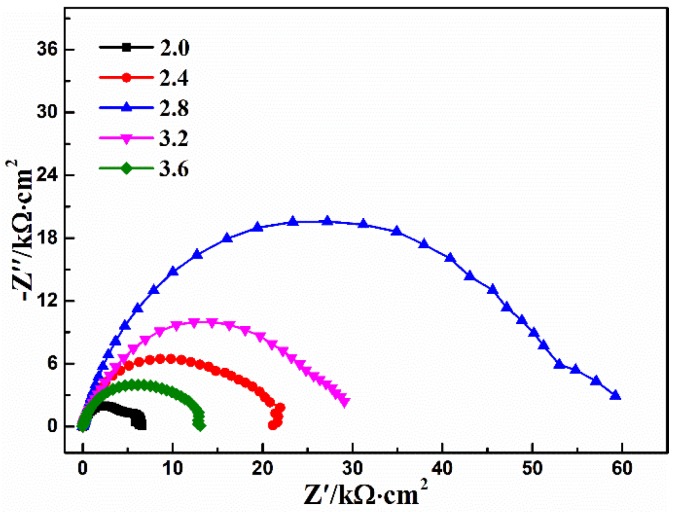
Experimental Nyquist plots of conversion coatings obtained from different pH values of Ca-P solution.

Figure 6 shows the EIS plots of the conversion coating obtained from different pH values of Ca-P solution adjusted by HCl or NaOH after fixing the concentration ratio of Ca(NO_3_)_2_·4H_2_O:NH_4_H_2_PO_4_ (5:3, pH = 2.8). The EIS fitting results are listed in Table 5. According to the fitting results, the Ca-P solution without any adjustment shows the best corrosion resistance, while the increase or decrease in pH value of the Ca-P solution weakens the protection of the conversion coating. The decrease in pH value of the Ca-P solution makes the corrosion reaction on the surface of the magnesium alloy dominate the whole reaction, while the increase in pH value of the Ca-P solution results in the appearance of precipitation, reducing the available content of coating-forming agent in the solution, which is not beneficial to the formation of conversion coating. On the other hand, when the pH of the Ca-P solution is above 2.8, the substrate etching rate may be too low to induce enough coating nucleation, and form the compact coating. Su et al. [27] also observe a similar phenomenon. Hence, the condition without any adjustment after simply fixing the Ca(NO_3_)_2_·4H_2_O:NH_4_H_2_PO_4_ concentration ratio (5:3) realizes the best protective effect for the AZ91D alloy, and the role of NH_4_H_2_PO_4_ acting simultaneously as a coating-forming agent and acidifying agent is also realized.

### 3.5. Stirring Rate

**Figure 7:**
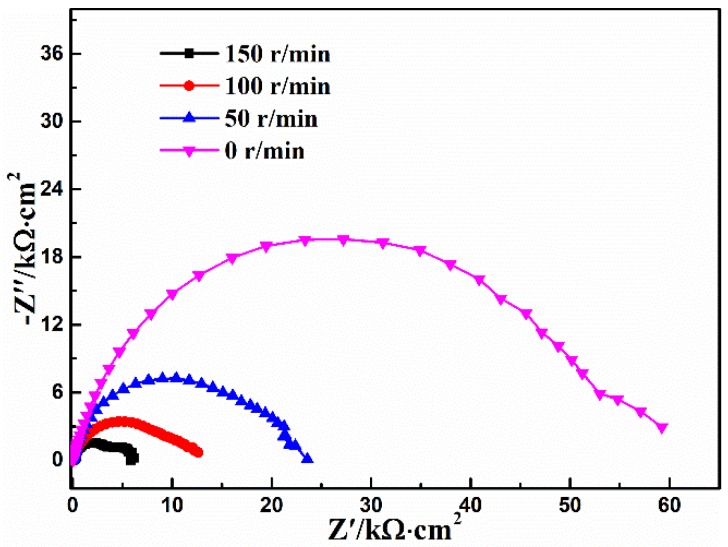
Experimental Nyquist plots of conversion coatings obtained from different stirring rates of Ca-P solution.

Figure 7 shows the effect of the stirring rate on the EIS characteristic of the calcium phosphate coatings, and the fitting results are listed in Table 6. According to Figure 7 and Table 6, it can be seen that the stirring rate exerts an important effect on the protection of the conversion coating. As long as the solution is stirred, the *R_coat_* decreases sharply. Meanwhile, the *R_coat_* gradually decreases as the stirring rate increases, and the conversion coating at the static condition presents the best corrosion resistance, i.e., stirring is not beneficial for the formation of the conversion coating. When the Ca-P solution is stirred, the solution close to the surface of the magnesium alloy is always acid. Hence the magnesium alloy keeps dissolving continuously, while the precipitation reaction does not easily occur on the surface of the magnesium alloy due to the quick diffusion of the species of deposition reaction caused by stirring, leading to the inferior protection of the conversion coating under dynamic conditions.

Based on the above discussions, the leaf-like coating on the AZ91D alloy provides the best corrosion resistance when the AZ91D is immersed in a Ca-P bath composed of 25 g/L Ca(NO_3_)_2_·4H_2_O and 15 g/LNH_4_H_2_PO_4_ for 20 min at a static condition of ambient temperature. From an electrochemical viewpoint, the corrosion resistance of calcium phosphate coating in this work is higher than that in some reported references [26,27,28,29,30,31]. Meanwhile, compared with those reported references [26,27,28,29,30,31], the preparation process of calcium phosphate coating in this work is also the simplest.

### 3.6. Coating Composition

**Figure 8:**
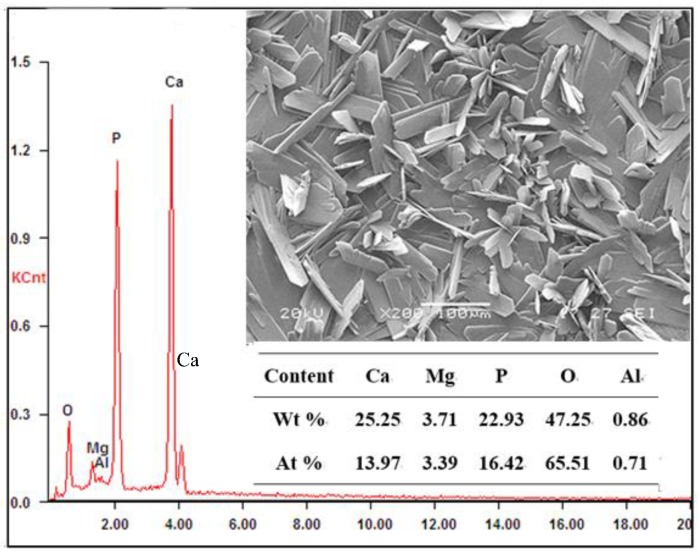
SEM micrograph of the Ca-P conversion coating and the correspondent EDS analysis.

**Figure 9:**
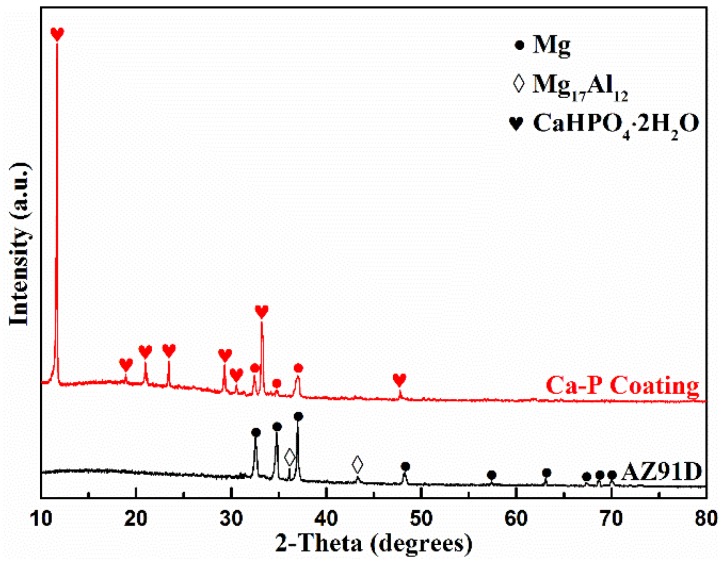
XRD patterns of the bare AZ91D sample and the AZ91D samples with Ca-P conversion coating.

Figure 8 shows the micrograph of the coating under the optimal conditions. It can be seen that the leaf-like coating uniformly and compactly covers the whole sample surface. The correspondent EDS analysis shows that the main elements of the coating are O, Ca and P. The atomic ratio of Ca:P:O listed in Figure 8 is approximately 1:1:4. Given the main composition of leaf-like Ca-P conversion coating in the reported literature [27,28,31,42], it can be inferred that CaHPO_4_·2H_2_O should be the main composition of the coating. Figure 9 shows the XRD patterns of the bare AZ91D alloy and the coating for further affirmation of the composition of the conversion coating. It is obvious that the substrate of the AZ91D alloy consists of α-Mg and β-Mg_17_Al_12_ with crystalline, while the coating is mainly composed of CaHPO_4_·2H_2_O (DCPD, JCDPS NO. 09-0077). In addition, the peaks of Mg substrate disappear, indicating that the DCPD coating basically covers the whole surface of the AZ91D alloy. Hence, the DCPD coating shows excellent protection for the AZ91D alloy.

### 3.7. Formation Mechanism

**Figure 10:**
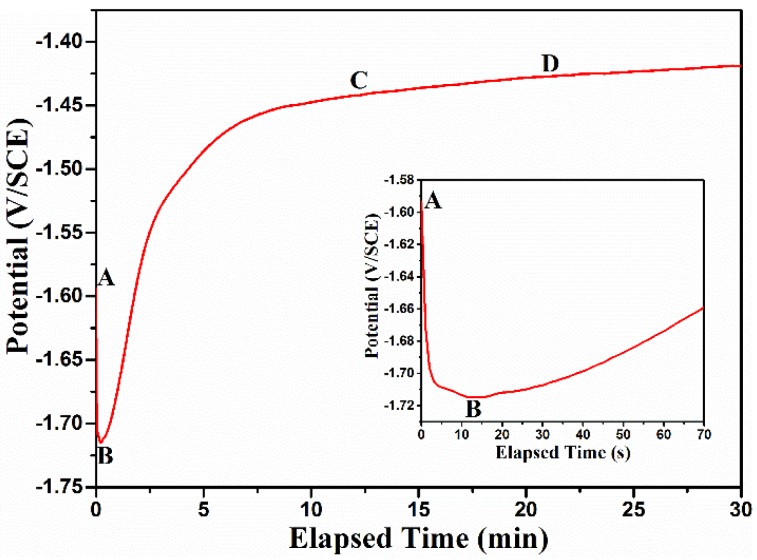
OCP curve during the conversion coating process.

For further discussion of the formation mechanism of the DCPD coating, OCP was measured as a function of time to monitor the deposition of conversion coatings [43], as shown in Figure 10. The OCP curve can be divided into three stages. In the initial several seconds (A–B), the OCP decreases quickly due to the activation of the surface of the Mg substrate and the dissolution of both the loose surface oxide and the Mg substrate in the acid Ca-P bath [30,41,43,44]. In the following stage (B–C), the OCP presents a sharp positive shift, indicating that the deposition of the calcium phosphate conversion coating begins, and then increasingly covers the surface. Therefore, the SEM shown in Figure 4 shows that the conversion coating gradually deposits on the AZ91D alloy from 5 min to 15 min, and thus both the diameter of the capacitive loop (Figure 3) and the corresponding *R_coat_* (Table 3) increase piece by piece. At the last stage (C–D), the OCP reaches a stable value, indicating that the deposition reaction at the solution/electrode interface has reached a stable state, and the dynamic equilibrium between the formation and dissolution of the DCPD coating has been established. Hence, the SEM micrograph of DCPD coating in 20 min shown in Figure 4 presents the best compactness, and thus can provide the best protection for the AZ91D alloy. With further prolongation of immersion time, the dynamic equilibrium between the formation and dissolution of DCPD coating may be destroyed, i.e., the coating dissolution rate may preponderate over the formation rate, and thus lead to the partial dissolution of the coating. Therefore, the coating at 25 min becomes sparse and thin, as shown in Figure 4, and the diameter of the capacitive loop at 25 min shown in Figure 3 reduces obviously, and thus cannot effectively prevent AZ91D from corroding. Additionally, due to the partial dissolution of the coating, the very small area of Mg substrate will be exposed to the calcium phosphate bath, and thus the cathodic hydrogen evolution may be slowly accelerated. Hence, OCP presents a sluggishly positive shift after the C–D stage [45].

**Figure 11:**
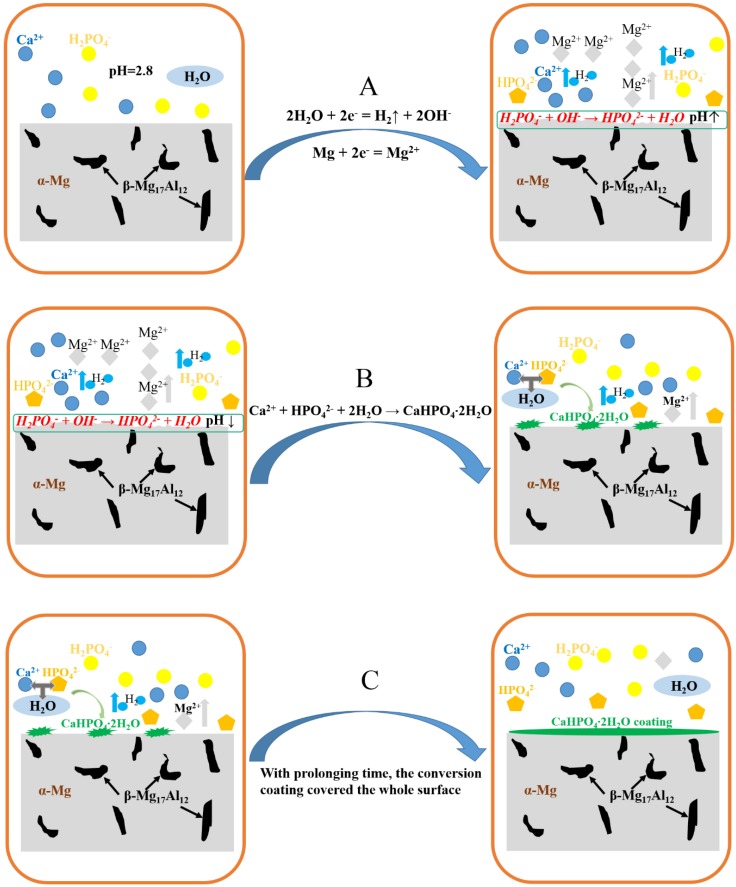
Formation mechanism of calcium phosphate conversion coating on AZ91D magnesium alloy: (**A**) corrosion and activation; (**B**) deposition of DCPD; (**C**) the whole coverage of DCPD coating.

Based on the above discussions, the formation mechanism of the DCPD coating on the AZ91D magnesium alloy is divided into three parts, A, B, and C, as shown in Figure 11. At the beginning, part A occurs at the surface of the AZ91D alloy, in which the anodic dissolution and cathodic hydrogen evolution result in the production of Mg^2+^, and the increase of the local pH close to the surface of the AZ91D alloy, respectively. The increase of OH^−^ will react with H_2_PO_4_^-^ to produce HPO_4_^2−^. As long as HPO_4_^2−^ ions exist in the Ca-P solution, part B will occur: the leaf-like DCPD coating deposits on the surface with some protection for the AZ91D alloy (Figure 3 and Figure 4). With the prolongation of time, the DCPD coating will gradually cover the whole surface of the AZ91D alloy as described in part C, which forms the compact DCPD coating with excellent corrosion resistance (Figure 3 and Figure 4). In this work, Ca(NO_3_)_2_·4H_2_O and NH_4_H_2_PO_4_ are selected as coating-forming agents to induce the formation of DCPD coating. Hence, the DCPD coating was obtained from a Ca-P bath with different Ca(NO_3_)_2_·4H_2_O:NH_4_H_2_PO_4_ concentration ratios, and can protect the bare AZ91D alloy from corrosion as shown in Figure 1. However, the different content of NH_4_H_2_PO_4_ in the Ca-P bath plays an important role in the corrosion resistance of the DCPD coating due to its acidifying effect. As the Ca(NO_3_)_2_·4H_2_O:NH_4_H_2_PO_4_ concentration ratio increases, i.e., the higher the concentration of coating-forming agent in the Ca-P bath, the pH of the Ca-P bath decreases. This decrease in pH can promote the initial coating nucleation due to the quicker activation of the Mg substrate, and cause a higher concentration of OH^−^ at the interface between metal and solution due to the easier hydrogen evolution [27,30], which is beneficial to the occurrence of part B, and thus forms a more compact DCPD coating. Therefore, the increase of NH_4_H_2_PO_4_ content remarkably improves the protective effect of DCPD coating for the bare AZ91D alloy. When the concentration ratio equals 5:3, the optimal pH (2.8) of DCPD formation with the best protective performance for the AZ91D alloy is realized. Yet, with the further increase of NH_4_H_2_PO_4_ content, there are precipitations existing in the bath, i.e., raw materials are wasted, the effective content of the coating-forming agent is decreased, and the pH decreases to a lower value. In this lower pH of the bath, the more rapid hydrogen evolution will retard the complete coverage of the DCPD coating, which leads to the decrease in corrosion resistance. Hence, the DCPD coating formed in the concentration ratio 5:3 of the Ca-P bath provides the better protective performance for bare AZ91D, compared to other concentration ratios.

## 4. Conclusions

In this work, a calcium phosphate coating was fabricated on the magnesium alloy AZ91D by very simple chemical conversion. The coating, with a leaf-like microstructure, is composed mainly of CaPO_4_·2H_2_O, and compactly covers the whole electrode surface, which can obviously strengthen the corrosion resistance of the AZ91D substrate. For obtaining the calcium phosphate coating, the AZ91D alloy was immersed in a Ca-P bath composed of 25 g/L Ca(NO_3_)_2_·4H_2_O and 15 g/L NH_4_H_2_PO_4_ (pH = 2.8, without any pH adjustment) for 20 min at a static condition of ambient temperature, which is very simple and suitable for popularization. Under such conditions, the role of NH_4_H_2_PO_4_ as simultaneously a coating-forming agent and acidifying agent is also realized. Furthermore, the formation mechanism of calcium phosphate conversion coating is proposed and the formation process is divided to three parts, among which the alkalization surrounding the magnesium substrate induced by corrosion activation, and inducing the production of HPO_4_^−^ plays an important role in the formation of the calcium phosphate conversion coating.

## Figures and Tables

**Table 1:** The fitting results of EIS shown in Figure 1a.

	*R_s_* (Ω·cm^2)^	*CPE_coat_* (μF·cm^−2^)	*n*_1_	*R_coat_* (kΩ·cm^2^)	*CPE_dl_* (μF·cm^−2^)	*n*_2_	*R_ct_* (kΩ·cm^2^)	*L_A_* (kΩ·cm^2^)	*R_A_* (H·cm^2^)	*χ*^2^ 10^−3^
none	5.86	-	-	-	12.7	0.91	1.26	4.84	888	4.8
5:1	7.88	12.4	0.90	2.87	707	0.82	1.38	-	-	0.8
5:2	12.4	16.1	0.82	18.3	-	-	-	-	-	4.1
5:3	17.8	13.2	0.80	56.3	-	-	-	-	-	4.0
5:4	12.3	17.1	0.80	29.2	-	-	-	-	-	2.9
5:5	11.9	15.2	0.82	25.6	-	-	-	-	-	3.5

**Table 2:** The fitting results of polarization curves shown in Figure 1b.

	*E_corr_* (V_SCE_)	*i_corr_* (μA/cm^2^)	*b_c_* (mV/dec)
none	−1.632	11.8	−159
5:1	−1.574	8.60	−167
5:2	−1.528	4.41	−166
5:3	−1.493	0.610	−140
5:4	−1.509	2.01	−171
5:5	−1.515	2.93	−156

**Table 3:** The fitting results of EIS shown in Figure 3.

Time (min)	*R_s_* (Ω·cm^2^)	*CPE_coat_* (μF·cm^−2^)	*n*_1_	*R_coat_* (kΩ·cm^2^)	*CPE_dl_* (μF·cm^−2^)	*n*_2_	*R_ct_* (kΩ·cm^2^)	*χ*^2^ 10^−3^
5	9.28	0.987	0.92	2.66	941	0.82	1.57	0.8
10	8.57	16.1	0.84	6.04	553	0.98	2.35	1.4
15	14.2	14.6	0.81	27.4	-	-	-	3.4
20	17.8	13.2	0.80	56.3	-	-	-	4.0
25	14.2	16.7	0.81	39.8	-	-	-	2.4

**Table 4:** The fitting results of EIS shown in Figure 5.

Temperature (°C)	*R_s_* (Ω·cm^2^)	*CPE_coat_* (μF·cm^−2^)	*n*	*R_coat_* (kΩ·cm^2^)	*χ*^2^ 10^−3^
Room temperature	17.8	13.2	0.80	56.3	4.0
20	15.6	11.6	0.81	54.8	4.2
30	15.0	13.1	0.80	56.7	3.4
40	15.6	12.1	0.82	60.2	4.0
50	11.9	20.9	0.79	23.6	1.7

**Table 5:** The fitting results of EIS shown in Figure 6.

pH	*R_s_* (Ω·cm^2^)	*CPE_coat_* (μF·cm^−2^)	*n*_1_	*R_coat_* (kΩ·cm^2^)	*CPE_dl_* (μF·cm^−2^)	*n*_2_	*R_ct_* (kΩ·cm^2^)	*χ*^2^ 10^−3^
3.6	8.7	18.7	0.81	10.7	345	0.99	2.40	0.9
3.2	11.3	20.9	0.77	29.1	-	-	-	0.9
2.8	17.8	13.2	0.80	56.3	-	-	-	4.0
2.4	11.5	15.6	0.81	20.1	-	-	-	3.9
2.0	8.66	14.2	0.85	4.96	522	0.96	1.56	1.3

**Table 6:** The fitting results of EIS shown in Figure 7.

Stirring Rate (r/min)	*R_s_* (Ω·cm^2^)	*CPE_coat_* (μF·cm^−2^)	*n*_1_	*R_coat_* (kΩ·cm^2^)	*CPE_dl_* (μF·cm^−2^)	*n*_2_	*R_ct_* (kΩ·cm^2^)	*χ*^2^ 10^−3^
150	6.95	14.5	0.84	4.02	448	0.96	1.93	1.5
100	9.01	23.6	0.80	8.97	810	0.87	3.32	0.5
50	11.1	18.3	0.82	21.3	-	-	-	3.5
0	17.8	13.2	0.80	56.3	-	-	-	4.0
